# Obesity and Nutritional Vulnerability in long COVID: A Neuroinflammatory and Cognitive Perspective

**DOI:** 10.1007/s13668-026-00730-y

**Published:** 2026-01-16

**Authors:** Cigdem Bozkir, Tugce Kartal, Busra Hokelek

**Affiliations:** 1 Faculty of Health Sciences, Department of Nutrition and Dietetics, Inonu University, Malatya, Türkiye; 2 Department of Nutrition and Dietetics, Cukurova University Hospital, Adana, Türkiye

**Keywords:** Long COVID, PASC, Obesity, Brain fog, Cognitive dysfunction

## Abstract

**Purpose of Review:**

To examine the interplay between obesity, nutritional vulnerability, and long COVID, with a particular focus on neuroinflammatory and cognitive outcomes. This review synthesizes emerging evidence on shared pathophysiological pathways and evaluates the therapeutic potential of dietary and weight management strategies.

**Recent Findings:**

Cognitive symptoms such as brain fog and memory deficits are among the most persistent and disabling features of long COVID. Obesity is associated with more severe manifestations through pathways involving chronic systemic inflammation, compromised blood-brain barrier integrity, and neuroimmune dysregulation. Concurrently, malnutrition and poor diet quality including low intake of antioxidants, omega-3 fatty acids, and micronutrients may impair neuroplasticity and delay recovery. Interventions such as Mediterranean and ketogenic dietary patterns, as well as structured weight loss programs, show promise in reducing inflammation and improving cognitive outcomes.

**Summary:**

Obesity and suboptimal nutritional status amplify the neurocognitive burden of long COVID through shared pathophysiological mechanisms. Integrated care models that incorporate metabolic screening, nutritional assessment, and individualized dietary interventions may improve recovery trajectories. Public health strategies that address food quality, obesity prevention, and equitable access to nutrition care are essential for long-term resilience in the post-COVID era.

## Introduction

The coronavirus disease 2019 (COVID-19) pandemic has left a lasting impact beyond the acute infection phase. A growing body of evidence now focuses on a new clinical entity: long COVID, also known as post-acute sequelae of SARS-CoV-2 infection (PASC). Long COVID (LC) is defined as the continuation or development of new symptoms 3 months after the initial SARS-CoV-2 infection, with these symptoms lasting for at least 2 months with no other explanation [1]. The World Health Organization now reports that approximately 6% of people infected with SARS-CoV-2 develop symptoms consistent with post COVID-19 condition [1]. The long-lasting complications of COVID-19 have recently been gaining increasing attention. The various clinical symptoms that affect many COVID-19 survivors are often collectively called LC. More than 200 LC symptoms have been identified, affecting most organs in the body [1, 2]. There is currently no gold standard for diagnosing LC; diagnosis is generally based on a compatible symptom history, persistence of symptoms over time, and self-report [2, 3].

Several factors are important risk factors for the development of LC. Chronic lung diseases (e.g., Chronic obstructive pulmonary disease) increase the risk by 32% and asthma by 41%, obesity, depression, female gender, and older age have been identified [4]. Additionally, not having received the COVID-19 vaccine, pre-existing comorbidities, and female gender were found to be the three strongest risk factors in another study [5]. A study by Barado et al. [6] shows a protective association, where vaccination is linked to a lower incidence of long-term COVID when adjusted for various factors, including gender, age, education, body mass index (BMI), physical activity, sleep, race, tobacco use, alcohol consumption, adherence to the Mediterranean diet, previous diseases, influenza vaccination, and healthcare worker status. Furthermore, healthy lifestyle habits prior to SARS-CoV-2 infection can significantly reduce the risk of developing LC. A study of 1,981 women in the United States (US), part of the Nurses’ Health Study II, examined six lifestyle factors: a healthy body mass index (18.5–24.9 kg/m²), not smoking, at least 150 min of moderate-to-vigorous physical activity per week, a healthy diet, moderate alcohol consumption (5–15 g per day), and adequate sleep duration (7–9 h per day) [7]. Individuals who maintained five or six of these habits had a 49% lower risk of LC-19. Specifically, a healthy body weight and adequate sleep independently reduced the risk [7]. Among its most troubling manifestations are cognitive symptoms colloquially termed “brain fog” which encompass difficulties in attention, memory, and executive function [8]. These cognitive issues appear to persist for weeks or months after the initial infection has resolved and are particularly concerning given their potential to reduce quality of life and occupational functioning [9].

Interestingly, COVID-19 may also disrupt appetite regulation, as evidenced by studies reporting elevated appetite-related hormones post-infection. Despite its limitations, this finding suggests that COVID infection could be a potential risk factor for obesity, independent of lifestyle changes caused by pandemic quarantine [10]. Obesity is a significant risk factor that worsens both acute COVID-19 and its long-term consequences. The chronic systemic and neurological inflammation observed in obesity [11] may underlie its role as a risk factor in the development of LC [12–14]. The systemic inflammation commonly seen in obesity often persists after SARS-CoV-2 infection [11, 12, 15]. This persistent inflammatory state may contribute to the chronicity of LC symptoms, such as fatigue, brain fog, muscle pain, and shortness of breath. Factors like depression, anxiety, and inactivity, frequently observed in individuals with obesity, may further exacerbate the neuropsychological effects of LC-19 [16].

While LC can affect individuals across the weight spectrum, recent research suggests that pre-existing obesity may predispose individuals to more severe or persistent symptoms [13–15]. At the same time, the role of nutritional status, including malnutrition or specific nutrient imbalances, remains an underexplored dimension. This review aimed to examine the complex interplay between LC, obesity, and nutrition, with an emphasis on the neuroinflammatory and cognitive outcomes of this triad.

## Conceptual Focus and Literature Mapping Strategy

This narrative review synthesizes the current evidence on the intersections between LC symptoms, obesity, and nutritional status, with a particular emphasis on cognitive dysfunction.

A targeted literature search was conducted in PubMed and Scopus databases covering the period from January 2021 to June 2025. The search strategy included terms such as “long COVID,” “post-acute COVID,” “obesity,” “body mass index,” “malnutrition,” “diet,” and “cognitive dysfunction.” Filters were applied to include only English-language, peer-reviewed articles focused on human subjects. The initial search yielded 76 records (45 from Scopus, 31 from PubMed). Articles were selected based on their relevance to the neurocognitive and nutritional dimensions of LC. After review, nine studies were duplicates, five involved children and adolescents, and twenty-four did not focus on the relationship between LC symptoms and obesity or nutritional consequences. Based on these search criteria, the current review focused on 38 studies.

Rather than aiming for exhaustive coverage, this review integrates key findings and mechanisms to highlight how obesity and nutritional vulnerabilities may shape post-COVID cognitive outcomes.

Several large-scale epidemiological studies have established a clear link between pre-existing obesity and an increased risk of developing LC and its neurological symptoms. To provide a clear evidence base for this review, we have summarized and critically appraised these key studies in Table 1.

**Table 1:** Epidemiological Studies on the Association Between Obesity and Long COVID

Study (Author, Year)	Study Design & Population	Key Epidemiological Finding	Strengths/Limitations
Wang S, et al. (2023)	Prospective cohort (1,981 female nurses from the US Nurses’ Health Study II)	Adherence to 5–6 healthy lifestyle factors (including BMI 18.5–24.9 kg/m²) was associated with a 49% lower risk of Long COVID (RR: 0.51).	S: Strong prospective design (shows lifestyle *before* infection); well-characterized cohort. L: Limited to a single sex (female) and mostly White (97.4%) healthcare professionals.
Subramanian A, et al. (2022)	Retrospective cohort; UK primary care database (*n* = 486,149 infected matched to 1.9 m controls).	Obesity was independently associated with an increased risk of Long COVID (aHR: 1.10). High BMI was linked to specific symptom clusters like breathlessness and fatigue.	S: Massive sample size; propensity score matching; controls for comorbidities. L: Relies on coded primary care data which may underestimate symptom prevalence.
Loosen SH, et al. (2022)	Cross-sectional study; German Disease Analyzer database (*n* = 50,402 COVID-19 patients).	Obesity (OR: 1.25) and lipid metabolism disorders (OR: 1.46) were identified as strong, age-independent risk factors for Long COVID.	S: Large, representative outpatient cohort; multivariate regression adjustment. L: Dependent on ICD-10 coding; lacks a non-COVID control group.
Nguyen KH, et al. (2024)	Cross-sectional; 2022 US Behavioural Risk Factor Surveillance System (*n* = 445,132).	Long COVID was significantly more prevalent among adults with overweight or obesity (aOR: 1.31) compared to those with normal weight.	S: Nationally representative US sample; assesses vaccination impact. L: Self-reported data subject to recall bias; cross-sectional design limits causal inference.
Bridger Staatz C, et al. (2023)	Longitudinal birth cohorts (UK); (NCDS; *n* = 7769, BCS70; n = 7168).	Earlier onset of obesity across the life course was associated with significantly higher odds of Long COVID in mid-life (OR: 2.15–3.01).	S: Unique life-course perspective; tracks BMI from childhood. L: Small number of Long COVID cases affecting statistical power for some sub-analyses.
Ronca DB, et al. (2025)	Systematic Review & Meta-Analysis (18 studies; *n* = 139,091 adults)	Excess weight is significantly associated with persistent neurological symptoms, including memory impairment (RR: 1.43) and sleep disturbances (RR: 1.31).	S: High-level evidence (meta-analysis); directly quantifies the risk for key neurological symptoms. L: Inherits the limitations of the primary studies it includes (e.g., varied definitions of Long COVID).
Schwartz CE, et al. (2024)	Cross-sectional Survey (656 participants with COVID in Spring 2023)	Latent Profile Analysis identified a high-burden group (40.9%) with severe multi-system symptoms; this group was characterized by higher BMI and financial hardship.	S: Provides recent (2024) data; identifies specific phenotypes (“faces”) of Long COVID. L: Cross-sectional (cannot prove causation); relies on self-reported data.

Abbreviations: *BMI* body mass index, *aHR* adjusted hazard ratio, *OR* odds ratio, *RR* risk ratio, *BRFSS* Behavioural Risk Factor Surveillance System, *NCDS* 1958 National Child Development Study, *BCS70* 1970 British Cohort Study. Notes: Definitions of long COVID/post-COVID-19 condition, follow-up duration, and symptom ascertainment vary across studies and are described in detail in the original publications (Refs 7, 12–15, 24, 61). Effect estimates (aHR, OR, RR) reported in the table are adjusted for key sociodemographic and clinical covariates as specified by each study. Overweight and obesity were generally defined using standard BMI cut-offs (overweight: 25.0–29.9 kg/m²; obesity: ≥30.0 kg/m²), unless otherwise stated in the original article

## Cognitive Manifestations of Long COVID

 Long COVID, or PASC, has emerged as a major public health concern affecting millions globally. A recent review summarizing multiple cohorts reported that approximately 60% of LC patients experienced brain fog and about 74% reported persistent fatigue [17]. These neurological manifestations often prove more debilitating than physical symptoms, fundamentally altering patients' quality of life and functional capacity [18].

A multicentre study of previously hospitalized COVID-19 survivors reported that 35.4% experienced post-COVID cognitive symptoms in the early months following infection [19]. More concerning, Northwestern University research published in 2024 demonstrates that neurological symptoms can persist for up to three years post-infection, with brain fog and fatigue being the most prevalent and impactful symptoms [20]. A systematic review evaluating 36 studies published between 2020 and 2023 found a permanent decline in executive function, memory, attention, and processing speed in long-term COVID-19 [17, 21]. A meta-analysis examining more than 4 million patients emphasized that symptoms such as brain fog, sleep disturbance, headache, and anxiety-depression were observed with high frequency in LC patients [22].

 Brain fog encompasses a constellation of cognitive symptoms including difficulty thinking or concentrating, confusion, forgetfulness, and feeling mentally slow [8]. Unlike traditional post-viral fatigue syndromes, LC brain fog appears to have distinct characteristics and may involve specific neurobiological mechanisms that are amplified in the presence of obesity.

The pathophysiological mechanisms underlying post-COVID cognitive dysfunction are not fully understood, but several mechanisms stand out. When SARS-CoV-2 enters the body, it binds to angiotensin-converting enzyme 2 (ACE2) and neuropilin-1 (NRP-1) receptors on endothelial and glial cells, initiating translocation of interleukin-6 (IL-6), interleukin-1 (IL-1), tumour necrosis factor (TNF), and migration of infected lymphocytes. Glial cell activation (microglia, astrocytes reactivation) occurs via pattern recognition receptors (PRRs) that detect the virus. Additionally, cytokines stimulate the hypothalamus-pituitary-adrenal (HPA) axis; neurotransmitter balances such as cortisol-dopamine-serotonin are disrupted, which negatively affects nerve conduction. Blood-brain barrier dysfunction occurs with disruption of tight junctions and entry of cytokines and immune cells into the central nervous system [18, 23]. This nervous system dysregulation, combined with strengthening of the systemic inflammatory response through neuro-immune feedback and the persistence of neuroinflammation, contributes to brain structural and functional disorders such as brain fog, anxiety, and memory disorders [18, 23].

While acute COVID-19 research extensively documented obesity's role in severe disease outcomes, the relationship between obesity and LC neurological symptoms has received limited attention. Emerging evidence suggests this oversight represents a critical gap in understanding LC pathophysiology. Increased BMI is closely associated with memory impairments and neurological symptoms (headaches, brain fog, myalgia, fatigue, and taste and smell disturbances) [24, 25]. Long-term neurological symptoms significantly reduce individuals' quality of life [11, 24]. Recent studies indicate that individuals with overweight experience more severe neurological effects of LC, particularly cognitive dysfunction and brain fog. This relationship appears independent of acute COVID-19 severity, suggesting unique mechanisms linking pre-existing metabolic dysfunction to persistent neurological symptoms [11, 16, 24].

In the United States, for example, over 42% of adults live with obesity [26]. Recent estimates suggest a wide variance in prevalence. A 2025 nationwide survey in the US reported that 7.2% of adults currently experience LC [27]. This contrasts with global meta-analyses reporting rates as high as 36% [5], a discrepancy likely driven by differences in study populations (general population vs. hospitalized patients) and diagnostic criteria.

##  Obesity as a Cognitive Amplifier in Long COVID 

 While direct experimental evidence in LC patients is limited, converging lines of evidence from observational studies, animal models, and mechanistic research suggest that obesity may amplify neurocognitive dysfunction through multiple interconnected pathways. Obesity is a state of chronic low-grade systemic inflammation, common in metabolic syndrome, type 2 diabetes, and cardiovascular conditions [28]. Adipose tissue, particularly visceral fat, acts as an endocrine organ, secreting inflammatory mediators like TNF-α, IL-6, adipokines, and free fatty acids, contributing to systemic inflammation [28–31].

 Individuals with obesity consistently show elevated circulating IL-6, TNF-α, and C-reactive protein (CRP) [28–30, 32, 33]. BMI positively correlates with plasma CRP, IL-1β, IL-6, and TNF-α, extending to central nervous system (CNS) inflammation with higher CSF IL-6 in individuals with overweight [32]. In acute COVID-19, patients with obesity exhibit higher initial and peak CRP, ESR, and d-dimer, suggesting a more exuberant inflammatory response [30]. While IL-6 and ferritin levels might be similar in acute severe cases, obesity's chronic inflammatory state provides a groundwork for worse outcomes [28, 30, 32].

 This chronic low-grade inflammation in obesity disrupts brain homeostasis, leading to oxidative stress, inflammation, altered hormones, insulin resistance, and blood-brain barrier (BBB) compromise [29, 34]. Obesity-induced neuroinflammation, characterized by elevated pro-inflammatory cytokines and reactive microglia/astrogliosis [35], primes the brain, making it less resilient and more susceptible to viral insults like SARS-CoV-2 [29, 34]. This preactivated state, with hyperactivated microglia producing neurotoxic chemicals, is hypothesized to amplify neurological consequences and cognitive dysfunction in LC [36, 37].

 During acute COVID-19, individuals with obesity consistently face increased risks for hospitalization, intensive care unit admission, mechanical ventilation, and higher mortality [28, 30, 31]. For instance, critical illness odds ratios are significantly higher in patients with obesity (e.g., OR 4.3 for BMI 30-40 kg/m², OR 6.3 for BMI >40 kg/m²). Mechanisms include dysregulated immune response, increased viral replication, and immunopathology, with adipose tissue potentially acting as a viral reservoir [30, 31].

 Beyond acute severity, obesity increases the likelihood of long-term neurological symptoms in LC [24, 38–40]. Excess weight is significantly associated with persistent depression, sleep disturbance, headache, memory issues, numbness, and vertigo [23]. Memory impairment and "brain fog" are common cognitive dysfunctions [38, 39]. Obesity-associated neuroinflammation can precede peripheral changes, directly impacting the CNS [41]. A negative correlation between BMI and cognitive function, and a positive correlation with inflammation (CRP, ESR), suggest a bidirectional interplay in LC [42].

 Obesity is associated with altered recovery trajectories and higher rates of chronic neurological and cognitive sequelae. The initial severe inflammatory response in individuals with obesity may lead to prolonged neuroinflammation. This potential self-reinforcing cycle, where higher BMI is associated with greater inflammation and worse cognitive function, highlights the potential for weight management interventions to improve cognitive recovery. Mitochondrial dysfunction, a neurodegenerative feature of COVID-19 neuropathy, is also more prevalent in individuals with metabolic syndromes like obesity [43].

 Obesity's chronic inflammatory milieu, with elevated IL-6, TNF-α, and CRP, compromises BBB integrity, facilitating immune cell infiltration and neuroinflammation [44]. Both obesity and high-fat diet (HFD) consumption are linked to reduced cognitive function via BBB disruption, showing increased permeability and decreased tight junction proteins [44, 45].

 Elevated inflammatory cytokines like IL-6 and TNF-α can cross the BBB, disrupting neural circuitry, inhibiting neurogenesis, and increasing permeability of brain microvascular endothelial cells [35, 44, 45]. Systemic inflammation can stimulate brain inflammatory responses, with pro-inflammatory cytokines directly influencing microglial activation [36]. BBB permeability increases during inflammation, exacerbating neurodegeneration by allowing peripheral immune cell infiltration [29].

 In animal models of obesity, peripheral monocytes have been observed infiltrating the CNS [44]. These cytokines also directly activate resident glial cells (microglia and astrocytes), establishing a primed neuroinflammatory state [35, 44]. HFD-induced gliosis promotes pro-inflammatory cytokine release [35], and activated microglia contribute to BBB dysfunction [36]. This pre-existing neuroinflammation may amplify SARS-CoV-2 neurological consequences [37].

 The interplay between BBB dysfunction and glial activation forms a reinforcing feedback loop: obesity-induced systemic inflammation compromises the BBB, allowing inflammatory factors and immune cells into the brain, which then activate glial cells, releasing more pro-inflammatory mediators that further disrupt the BBB [36, 44, 45]. This potential self-perpetuating cycle is hypothesized to render the brain more vulnerable and less capable of recovery, possibly amplifying neurological damage and cognitive dysfunction in LC [37, 41].

 Beyond inflammation, obesity's compounded vascular and metabolic burdens create a hostile neurovascular environment. Obesity is linked to macro- and microvascular endothelial dysfunction, a risk factor for cognitive impairment. Endothelial dysfunction disrupts blood flow, reduces vascular tone, and impairs the BBB, partly due to reduced nitric oxide (NO) bioavailability and impaired neurovascular coupling [45]. Dysregulation of cerebral blood flow (CBF) in obesity contributes to oxidative stress in the brain [34].

 Obesity is strongly linked to insulin resistance, affecting metabolic organs and interfering with insulin signalling [28, 46, 47]. Brain insulin resistance contributes to metabolic disease [46]. Obesity is associated with cognitive decline through multiple pathways, including impaired metabolic and neurological function [48]. More specifically, reduced brain glucose transport capacity has been observed in individuals with obesity [49]. Metabolic dysfunction is a risk factor for severe acute COVID-19 and may predispose to PASC, with hyperglycaemia predicting adverse outcomes [47].

 These vascular, metabolic, and inflammatory burdens synergistically interact. Obesity induces a "feed-forward cycle" culminating in endothelial cell dysfunction and cognitive impairment [45]. Inflammation exacerbates insulin resistance, worsening endothelial dysfunction, compromising the BBB, and fuelling neuroinflammation [28, 45]. This multi-systemic dysregulation compromises the brain's resilience and recovery capacity [40, 43, 47, 48]. Metabolic syndrome, including obesity, increases viral infection severity and mortality, and dysregulates immune response [50]. Impaired brain energy metabolism, evidenced by reduced cerebral glucose transport capacity [49], may increase susceptibility to cognitive decline in individuals with obesity. Mitochondrial dysfunction is also more prevalent in individuals with metabolic syndromes like obesity [43]. Consequently, individuals with obesity are prone to more severe and persistent cognitive deficits in LC, including brain fog, memory issues, and impaired executive functioning [24, 38, 43, 48].

##  Nutritional Status and Recovery Outcomes

 While metabolic dysfunction plays a critical role, it is often inextricably linked to diet quality. Nutritional deficits, including both undernutrition and poor diet quality, may independently contribute to prolonged cognitive symptoms. Post-COVID malnutrition is associated with a variety of factors. Catabolic stress due to systemic inflammation, metabolic abnormalities, and decreased respiratory function are the primary causes [51]. Gastrointestinal symptoms, taste and smell disorders, psychiatric symptoms, fatigue, and muscle weakness associated with long-term COVID may contribute to malnutrition in the long term [51]. In a study in the USA, which included 92 people aged 18-90, it was determined that most of the participants did not consume fruits and vegetables in accordance with national recommendations, 48% were at risk of malnutrition, only 39% provided the optimal protein intake specific to the population, and the distribution of protein intake across meals was unbalanced (only 3% of the participants achieved the recommended level of protein intake at each meal, and 30% did not reach the recommended intake at any meal) [52]. Emerging evidence suggests that diets low in antioxidants, omega-3 fatty acids, or B vitamins may be associated with impaired neuroplasticity and cognitive decline, both in aging and post-viral contexts [53]. Omega-3 fatty acids (especially eicosapentaenoic acid and docosahexaenoic acid) are thought to support immune function by counteracting inflammation. Specific micronutrients such as vitamin D, zinc, and selenium are critical to immune function and neural repair. While several studies have documented nutrient deficiencies in LC populations [52, 53], others have emphasized the importance of evaluating such deficits through targeted interventions [54].

 In a study conducted in Scotland, 234 individuals with long-term COVID symptoms for ≥12 weeks and a BMI >27 kg/m² (>25 kg/m² for participants of South Asian descent) were randomly divided into two groups: 118 in the intervention group and 116 in the control group [55]. The intervention group received a low-calorie diet containing approximately 850 kcal per day for 12 weeks. At the end of the 12 weeks, a controlled diet model tailored to individual needs was implemented under the supervision of a dietitian. After 6 months, an average weight loss of 10.3 kg was observed in the intervention group and an average weight loss of 0.7 kg in the control group. Significant improvements were also reported in individual symptoms such as blood pressure, quality of life, fatigue, shortness of breath, anxiety, and depression in the intervention group. While cognitive function was not the primary endpoint, the reduction in these neuropsychological symptoms suggests that nutritional weight management may yield co-benefits for neurocognitive recovery, though further research is needed to confirm this [55]. A study investigated the effectiveness of two dietary models (ketogenic and Mediterranean) applied to individuals with obesity after COVID-19. The ketogenic diet is a high-fat diet where carbohydrate intake is restricted to less than 50 grams per day. The Mediterranean diet, on the other hand, is low in saturated fat but rich in fibre and monounsaturated and polyunsaturated fatty acids, with high plant-based foods (whole grains, fruits, vegetables, legumes, and oilseeds) and fish, moderate dairy products and alcohol, and low red meat. The ketogenic diet, implemented in the first phase, accelerates weight loss thanks to its low carbohydrate and high fat content, reduces obesity-related inflammation, and modulates the immune system. During this process, the anti-inflammatory effects of ketone bodies (especially β-hydroxybutyrate) reduce proinflammatory cytokines and regulate cellular energy balance. This plays an important role in combating hypoxemia and systemic inflammation that occur after COVID-19 [56]. In a pilot study by Barrea et al. [56], a biphasic approach was proposed where the ketogenic diet is implemented in the first phase to achieve sustainable health goals. This transition ensures both the retention of metabolic gains and allows the individual to maintain their nutritional habits over the long term. Rich in olive oil, fruits and vegetables, whole grains, and fish, the Mediterranean diet strengthens the immune system thanks to antioxidants, polyphenols, and omega-3 fatty acids, reduces oxidative stress, and contributes to systemic healing by regulating the gut microbiota. It may also limit SARS-CoV-2 entry into cells by regulating ACE2 expression. This holistic approach is thought to be an effective nutritional strategy for PASC management, particularly in individuals with obesity. Integrating physical activity into this process can support both physical and metabolic recovery [56]. A study conducted in Spain with 305 adults diagnosed with long COVID-19 according to WHO criteria found that high adherence to the Mediterranean diet was negatively associated with BMI, waist circumference, and components of metabolic syndrome, and positively associated with HDL levels. Adherence to the Mediterranean diet is thought to be beneficial for cardiovascular health, metabolic deterioration, and the reduction of inflammation [57]. A scoping review of 50 studies, 27 interventions and 23 observational studies, evaluated the effects of micronutrients (e.g. vitamins D and K2), amino acids (e.g. L-arginine), multinutrient formulations, microbiota-targeted therapies (e.g. probiotics, symbiotic), nutritional status, diet quality, and dietary approaches such as the Mediterranean diet. Approximately 76% of studies reported improvements in LC symptoms such as fatigue, mood disorders, physical function, and markers of inflammation [53]. It is also emphasized that diets rich in fibre and antioxidants can reduce the development of PASC by improving the intestinal microbiota [58].

 It should be noted that while these nutritional and weight management interventions show promise for metabolic markers and general symptom improvements, direct evidence specifically linking these interventions to cognitive and neuroinflammatory outcomes in LC remains limited. Most studies to date have focused on fatigue, quality of life, and metabolic parameters, with cognitive function rarely measured as a primary endpoint [55]. Further investigation through targeted, controlled trials with validated neurocognitive assessments and inflammatory biomarkers is needed to establish whether nutritional interventions can directly improve brain fog, memory deficits, and neuroinflammation in LC populations.

 These findings are summarized in Table 2, which outlines key nutritional interventions evaluated in individuals with LC and highlights their clinical impact, target population, and proposed mechanisms of action.

**Table 2:** Summary of key nutritional interventions and their impact on long COVID symptoms

Study (Author, Year)	Intervention Type	Study Population	Key Findings	Proposed Mechanisms
Combet et al., 2025	Remotely delivered low-calorie diet (850 kcal/day for 12 weeks, then tailored)	234 Long COVID patients with overweight/obesity (BMI > 27 kg/m²)	Average weight loss of 10.3 kg; Significant improvements in blood pressure, quality of life, fatigue, shortness of breath, anxiety, depression.	Weight management, reduction of systemic inflammatory burden.
Barrea et al., 2022	Ketogenic diet (first phase) followed by Mediterranean diet (second phase)	Individuals with obesity after COVID-19	Accelerated weight loss, reduced obesity-related inflammation, modulated immune system, sustained health goals.	Ketone bodies (β-hydroxybutyrate) anti-inflammatory effects, regulation of cellular energy balance; Antioxidants, polyphenols, omega-3s, gut microbiota regulation, ACE2 expression modulation.
Suárez-Moreno et al., 2025	Adherence to Mediterranean diet (observational)	305 adults with Long COVID-19	High adherence negatively associated with BMI, waist circumference, metabolic syndrome components; positively associated with HDL levels.	Beneficial for cardiovascular health, metabolic deterioration, reduction of inflammation.
Bigman et al., 2025	Various micronutrients, amino acids, multi nutrient formulations, microbiota-targeted therapies, diet quality, dietary approaches (scoping review)	Long COVID patients (across 50 studies)	Approximately 76% of studies reported improvements in fatigue, mood disorders, physical function, and markers of inflammation.	Broad range of mechanisms depending on intervention, including immune modulation, oxidative stress reduction, gut microbiota improvement.
Reyes et al., 2024	Diets rich in fibre and antioxidants (review)	General Long COVID context	Reduced development of PASC.	Improvement of intestinal microbiota.

##  The Obesity-Nutrition-Cognition Axis

 The co-existence of obesity and poor nutritional status constitutes a"syndemic" a complex, interacting set of health conditions that compound each other's effects particularly evident in the context of LC. This syndemic framework has been increasingly recognized to explain the synergistic interaction between metabolic dysfunction and nutritional deficits, both of which can independently and jointly compromise neurocognitive resilience [38].

 increased levels of circulating cytokines such as IL-6, TNF-α, and CRP, which can cross the BBB and trigger neuroinflammatory responses [44]. At the same time, poor nutritional intake such as diets lacking in antioxidants, omega-3 fatty acids, and essential micronutrients further diminishes the brain's ability to counteract oxidative stress and support neurotransmission [29, 45, 53]. These biological insults are associated with alterations in several interconnected pathways: endothelial dysfunction is linked to impaired cerebral perfusion [45]; insulin resistance is associated with disrupted neuronal energy metabolism [46, 48]; oxidative stress has been linked to mitochondrial damage [43]; and gut dysbiosis enhances systemic inflammation, ultimately weakening the BBB and promoting cognitive decline [44, 53]. The convergence of these mechanisms is particularly detrimental in LC, where sustained viral and inflammatory insults already challenge brain function [36, 40].

 Emerging therapeutic strategies aim to address these multifaceted pathways. Resveratrol, which is polyphenolic compound found in grapes and red wine, has been shown to modulate the gut microbiota, increase beneficial bacterial strains (e.g., Lactobacillus, Bifidobacterium), reduce the Firmicutes/Bacteroidetes ratio, and suppress systemic inflammation through NF-κB inhibition [59]. Furthermore, its antioxidant capacity helps preserve the integrity of brain capillary endothelial cells and reduce BBB permeability, thus mitigating neuroinflammation and oxidative stress [53–59].

 Conceptualizing the obesity-nutrition-cognition axis enables a deeper understanding of how coexisting metabolic and nutritional stressors shape the trajectory of cognitive symptoms in LC. Recognizing and intervening in this axis early may offer a critical opportunity to prevent long-term disability in affected populations.

 The mechanistic flow of these complex interactions is detailed in Table3, which maps this cascade from initial systemic drivers (obesity, malnutrition) to the final, converged outcome of neuroinflammation and cognitive dysfunction.

**Table 3:** A mechanistic flow from systemic drivers to long COVID cognitive dysfunction

Mechanistic Step	Contribution from Obesity	Contribution from Malnutrition/Poor Diet
(1) Chronic Systemic Inflammation	Elevated IL-6, TNF-α, & CRP from adipose tissue; primes the body for a hyper-inflammatory response [40, 44].	Deficiencies in anti-inflammatory nutrients (e.g., omega-3s, antioxidants); pro-inflammatory diet [53].
(2) Endothelial Dysfunction	Impaired nitric oxide bioavailability and increased vascular inflammation due to metabolic stress, compromising blood vessel integrity [45].	Deficiencies in endothelial-supporting nutrients (e.g., vitamin D, L-arginine) [53].
(3) Metabolic Dysregulation (Insulin Resistance)	Impaired glucose uptake in peripheral tissues and brain; has been linked to advanced glycation end products (AGEs) [29, 46, 49].	High intake of sugars; micronutrient deficiencies affecting glucose metabolism [53].
(4) Gut Microbiota Dysbiosis	Altered gut microbiota composition promotes a pro-inflammatory state and increases gut permeability (“leaky gut”) [59].	Lack of fibre and prebiotics; imbalance of beneficial bacteria; nutrient malabsorption [53, 59].
(5) Mitochondrial Dysfunction & Oxidative Stress	Increased reactive oxygen species (ROS) from stressed adipose tissue and hypermetabolism; impaired antioxidant defences [43].	Deficiencies in antioxidant vitamins (C, E) and minerals (selenium, zinc) needed to combat ROS [43, 53].
(6) Blood-Brain Barrier (BBB) Dysfunction	The combined systemic assault from inflammation [44] and endothelial dysfunction [45] compromises tight junctions, making the BBB permeable.	Micronutrient deficiencies weaken BBB integrity; inflammation from gut dysbiosis adds to the systemic load [36, 53].
(7) Neuroinflammation & Cognitive Impairment	Inflammatory cytokines and immune cells cross the compromised BBB [36]. Brain insulin resistance [46] and mitochondrial dysfunction [43] are associated with impaired neuronal energy metabolism.	Impact: Microglial activation, impaired neurotransmitter balance, reduced neuroplasticity, and direct neuronal damage, manifesting as “brain fog,” memory deficits, and fatigue [17, 38, 40].

##  Clinical Implications and Care Models

 The multisystemic and multifactorial nature of LC calls for a holistic, integrated clinical care model that encompasses not only virological management but also metabolic, nutritional, and neurocognitive dimensions. Given the strong association between obesity and persistent LC symptoms, especially cognitive dysfunction, routine nutritional screening and BMI monitoring should be standard components of post-COVID clinical follow-up [24, 55].

 Dietary interventions such as the Mediterranean diet or the phased ketogenic-to-Mediterranean dietary approach have demonstrated potential in improving both metabolic health and cognitive symptoms. The Mediterranean diet, rich in anti-inflammatory nutrients, fibre, polyphenols, and omega-3 fatty acids, supports gut microbiota diversity and improves endothelial function both critical for brain health. Meanwhile, the ketogenic diet, known for its capacity to induce ketosis and modulate immune responses, may be especially beneficial in reducing neuroinflammation in the early post-COVID phase [56].

 Preventive strategies should focus on individuals with obesity, malnutrition, or both, who are at significantly elevated risk for long-term cognitive and physical impairment. Observational studies have shown that high adherence to healthy dietary patterns is associated with lower BMI, improved inflammatory profiles, and better cognitive outcomes among LC patients [53, 57].

 Moreover, multidisciplinary care teams that include registered dietitians, clinical psychologists, rehabilitation specialists, and primary care providers are essential for personalized recovery plans. Integrating nutrition-based therapies with cognitive rehabilitation and physical activity can create a synergistic effect, improving neuroplasticity, reducing fatigue, and enhancing quality of life. These integrative care models offer a cost-effective, sustainable strategy to manage the long-term burden of LC, particularly in vulnerable populations with existing metabolic or nutritional risks.

##  Public Health and Research Directions

 A 2022 study in the US estimated that 7.2% of adults had experienced LC in the general population [27]. This contrasts with higher estimates (up to 30%) reported in other North American studies [5], a discrepancy likely due to differences in study populations (e.g., hospitalized vs. non-hospitalized) and diagnostic definitions.

 From a healthcare perspective, individuals diagnosed with long COVID-19 visited a healthcare facility an average of 30 times per year, a number significantly higher than the control group [60]. This increase was particularly pronounced in women, individuals with asthma or mental health issues, and those with prior hospitalizations. Individuals diagnosed with LC-19 were found to generally have a greater healthcare burden and significantly increased healthcare costs. This suggests that public health policies should allocate resources to the prevention and treatment of LC-19 [60–62].

 Future studies should explore how diet and adiposity modulate LC trajectories via immune, endocrine, and neural pathways. Longitudinal cohorts with biomarker data are needed to disentangle causality.

 Public health policies should address food insecurity and obesity prevention as part of post-COVID recovery planning. Nutrition literacy and access to nutrient-rich foods are key components in building long-term resilience. Prioritizing access to LC treatment, especially for individuals with overweight/obesity, establishing gender-specific approaches as LC and obesity are more common in women, adopting treatment approaches targeting weight management and inflammation reduction, and establishing community-based physical activity and nutrition recommendations should be the main approaches [4, 5, 63, 64].

 As a narrative review, this manuscript is a descriptive synthesis of the literature and is subject to certain limitations. The absence of a formal search protocol means the selection of articles may reflect some author-specific bias, and the heterogeneity of included studies makes direct comparisons challenging. Most of the evidence linking obesity to LC cognitive issues comes from observational research, which shows associations rather than clear cause and effect. Specifically, the studies connecting obesity to a higher risk of LC are mostly snapshots in time or look back at past data; this means they cannot prove which condition came first or rule out that the illness itself led to weight changes. Furthermore, the biological pathways described such as brain inflammation, leaky blood-brain barriers, and insulin resistance are largely based on animal studies or general obesity research rather than direct testing in LC patients. While diet and nutrition studies show improvements in metabolism and general health, very few have specifically measured brain inflammation or thinking skills in people with LC, and it is often hard to separate the independent effects of obesity from those of common comorbidities. Therefore, this review should be viewed as a conceptual framework that highlights plausible hypotheses and underscores the need for more rigorous, interventional studies to validate these proposed mechanisms establish causal relationships.

##  Conclusion

 The relationship between obesity and LC, particularly its neurocognitive manifestations, represents a critical and evolving area of public health concern. While obesity's role as a risk factor for severe acute COVID-19 outcomes is well-documented, this review highlights its significant, though still emerging, influence on the persistent neurological symptoms, notably brain fog and cognitive dysfunction, that characterize LC. This narrative review, synthesizing post-2021 literature, suggests that pre-existing obesity and nutritional vulnerabilities may significantly amplify the risk and severity of neurocognitive manifestations. The underlying mechanisms appear to be multifactorial and interconnected, involving the exacerbation of neuroinflammatory cascades, compromise of blood-brain barrier integrity, and the critical role of specific nutrient deficiencies and poor diet quality. The chronic low-grade systemic inflammation characteristic of obesity seems to prime the central nervous system, making it more susceptible to the neuroinflammatory processes initiated by SARS-CoV-2 infection. Furthermore, the co-existence of obesity and poor nutrition forms a "syndemic," where shared pathological pathways including chronic inflammation, insulin resistance, and oxidative stress may synergistically impair neurocognitive recovery.

 The evidence presented suggests the potential of targeted nutritional and weight management interventions as viable, non-pharmacological therapeutic strategies. Studies indicate that programs and specific dietary patterns can improve not only metabolic markers but also cognitive function, fatigue, and overall quality of life in affected individuals. This understanding may necessitate a shift toward integrated care models that incorporate nutritional screening and metabolic assessment into standard LC care. From a public health perspective, the healthcare burden and economic costs associated with LC underscore the urgent need for equitable resource allocation and targeted interventions. Addressing the obesity epidemic and improving nutritional literacy appear to be critical components of national public health preparedness. Ultimately, a holistic approach that integrates obesity prevention and treatment into public health strategies seems essential for improving the quality of life for millions affected by LC and for building more resilient healthcare systems.

## Key References

- Bigman G, Rusu ME, Shelawala N, Sorkin JD, Beamer BA, Ryan AS. A Comprehensive Scoping Review on Diet and Nutrition in Relation to Long COVID-19 Symptoms and Recovery. Nutrients. 2025;17(11):1802. https://doi.org/10.3390/nu17111802.
  - ◦ The aim of this scoping review was to provide a broad overview of the existing literature on the relationship between diet, nutrition, and LC. It found a significant association between various dietary patterns and the severity and persistence of symptoms, highlighting the potential for nutritional interventions to aid in recovery.
- Combet E, Haag L, Richardson J, et al. Remotely delivered weight management for people with LC and overweight: the randomized wait-list-controlled ReDIRECT trial. Nat Med. 2025;31(1):258-266. https://doi.org/10.1038/s41591-024-03384-x.
  - ◦ This randomized controlled trial evaluated the impact of a structured weight management program on LC symptoms in individuals with overweight. It demonstrated that the intervention led to significant improvements in primary symptoms, fatigue, and quality of life, providing strong evidence for weight management as a therapeutic strategy.
- Ronca DB, Mesquita LO, Oliveira D, et al. Excess weight is associated with neurological and neuropsychiatric symptoms in post-COVID-19 condition: A systematic review and meta-analysis. PLoS One. 2025;20(5):e0314892. https://doi.org/10.1371/journal.pone.0314892.
  - ◦ This systematic review and meta-analysis aimed to assess the link between excess weight, and the neurological and neuropsychiatric symptoms of post-COVID-19 condition. The findings revealed a robust and statistically significant association, confirming that excess weight is a major risk factor for these specific symptoms and reinforcing the role of obesity-related inflammation.

## Data Availability

No datasets were generated or analysed during the current study.

## Abbreviations

ACE: 2 Angiotensin-converting enzyme 2
BMI: Body mass index
CDC: Centers for Disease Control and Prevention
CNS: Central nervous system
CRP: C-reactive protein
COVID: Coronavirus disease
HFD: High-fat diet
HPA: Hypothalamus-pituitary-adrenal
IL-1: Interleukin-1
IL-6: Interleukin-6
LC: Long COVID
NRP-1: Neuropilin-1
PASC: SARS-CoV-2 infection
PRRs: Pattern recognition receptors
TNF: Tumor necrosis factor
US: United States
WHO: World Health Organization
